# Supplementary material for: Genetic Diversity of Dengue Vector Aedes albopictus Collected from South Korea, Japan, and Laos
Source: Insects. 2023 Mar 20;14(3):297. doi: 10.3390/insects14030297 (PMC10051289; doi:10.3390/insects14030297)
Supplement: Supplementary file 1 [file insects-14-00297-s001.zip › insects-2233716-supplementary.pdf]

Table S1 Microsatellite information used in the study of *Ae. albopictus.*

Locus	GenBank accession number	Primer sequence (5'-3')	Fluorescent dyes	Size range (bp)	References
AealbB51	DQ366023	F:TCCACGTGGTATAACTCTGA<br>R: GTAGTTGTCCAATTAACATCG	NED	141	[43]
AealbB52	DQ366024	F:GGGTCTAGAAGTAATAGCGATG<br>R: GCATTCTTTGCTTCTGTTTGC	FAM	173	[43]
AealbF3	DQ366027	F:CTCGTGAGTACGTTCCGTGA<br>R: AGGGAAACAAGGACTTCATCA	VIC	247	[43]
AealbD2	DQ366021	F:GAATCCCACACAGCGTCTTT<br>R: GGTCGCTTGACACCTTGAAT	VIC	238	[43]
Alb-di-4	KF146971	F:TGGCGACCTATTATACCCGC<br>R: CAACTCGTTCCTTGACCGTG	FAM	166–200/179	[41]
Alb-di-6	KF146972	F:TCTTCATCTACGCTGTGCTC<br>R: GACGCCAATCCGACAAAGTC	PET	268-290/255	[41]
Alb-tri-3	KF146973	F:AGATGTGTCGCAATGCTTCC<br>R: GATTCGGTGATGTTGAGGCC	NED	123-153/117	[41]
Alb-tri-18	KF146975	F:ACACAATTGCCGTTCAGCTC<br>R: CGTCTAATAGCTCCGGTCCC	VIC	250-280	[41]
Alb-tri-20	KF146976	F:GTGCCGTTGATCATCCTGTC<br>R: TCCAGCACCGTGAGTAATCC	FAM	165-201	[41]
Alb-tri-25	KF146978	F:CCAACCAACAACCCAGGAAC<br>R: TACGATGCGCAACCATCATC	PET	257-278	[41]
Alb-tri-41	KF146980	F:GATCGATTTGGGAGCTTCTG<br>R: GAACCTCTTCTCGCTTGGCT	NED	134-155	[41]
Alb-tri-45	KF146982	F:TTTCAGCTCGGTGTTATGGC<br>R: TGATGTTGATGATGATGACTACGA	NED	120-150	[41]
Alb-tri-46	KF146983	F:TTCACAACATACGGAATCGC<br>R: GGTCCGGTGTAATAGCCTCC	FAM	158-192	[41]
Aealbmic8	KP859598	F: TTGTTGTTCGGTTGTTGTTTG<br>R: CGGGTTCCAACTATGTACGA	PET	453-468	[42]
Aealbmic21	KP859611	F: CCCTACAGCCCTGATTGAGA<br>R: CGAGTTGGGATGTGTGATTG	VIC	212-232	[42]
Aealbmic23	KP859613	F: AACGGAGCGGAGTCGATTAT<br>R: CTACTACCCGCTGCCTTCTG	PET	331-354	[42]

Table S2 Haplotype distribution and GenBank accession number of *Ae. albopictus* used in this study

Haplotype	Locality																					Total
	WJ-I	WJ-II	YC	YS	AY-II	AY-I	CC	CY	DJ	GC	GJ	GW	GY	JD	JJ	SC	SE	YJ	YE	TJ	VL	
H1	12	17	5	6	3	1	7	2	8	5	0	2	1	2	26	11	2	3	2			115
H2	1																					1
H3	1																					1
H4	3			1												1						5
H5	1																					1
H6		1																				1
H7				2																		2
H8				1																		1
H9				1																		1
H10				1																		1
H11					1																	1
H12						1																1
H13						1																1
H14						1																1
H15										1												1
H16										1												1
H17											3											3
H18											2											2
H19													1									1
H20														2								2
H21														1						29		30
H22															1							1
H23															1							1
H24																1						1
H25																	1					1
H26																				5		5
H27																				1		1
H28																					1	1
H29																					1	1
H30																					4	4
H31																					15	15
H32																					1	1
H33																					1	1
H34																					1	1
H35																					1	1
H36																					1	1
H37																					1	1
H38																					1	1

Locality name	CO1	ND5
Wonju (2017)	MW526509-MW526526	MW526720-MW526737
Wonju (2020)	MW526527-MW526544	MW526738-MW526755
Yeoncheon	MW526545-MW526549	MW526756-MW526760
Yangsan	MW526550-MW526561	MW526761-MW526772
Anyang (2020)	MW526562-MW526565	MW526773-MW526776
Anyang (2018)	MW526566-MW526569	MW526777-MW526780
Chuncheon	MW526570-MW526576	MW526781-MW526787
Cheongyang	MW526577-MW526578	MW526788-MW526789
Daejeon	MW526579-MW526586	MW526790-MW526797
Gwacheon	MW526587-MW526593	MW526798-MW526804
Geoje	MW526594-MW526598	MW526805-MW526809
Gwangju	MW526599-MW526600	MW526810-MW526811
Gyeongju	MW526601-MW526602	MW526812-MW526813
Jeung-do	MW526603-MW526607	MW526814-MW526818
Jeonju	MW526608-MW526635	MW526819-MW526846
Sokcho	MW526636-MW526648	MW526847-MW526859
Seoul	MW526649-MW526651	MW526860-MW526862
Yeoju	MW526652-MW526654	MW526863-MW526865
Yeosu	MW526655-MW526656	MW526866-MW526867
**Tokyo Japan,**	MW526657-MW526691	MW526868-MW526902
**Vientiane, Laos,**	MW526692-MW526719	MW526903-MW526930

Abbreviations: WJ-I= Wonju (2017), WJ-II= Wonju (2020), YC= Yeoncheon, YS= Yangsan, AY-I= Anyang (2018), AY-II= Anyang (2020), CC= Chuncheon, CY= Cheongyang, DJ= Daejeon, GC= Gwacheon, GJ= Geoje, GW= Gwangju, GY= Gyeongju, JD= Jeungdo, JJ= Jeonju, SC= Sokcho, SE= Seoul, YJ= Yeoju, YE= Yeosu, TJ= Tokyo, Japan, VL= Vientiane, Laos

Table S3 Pairwise differentiation (F_{ST}) for concatenated sequences of the mitochondrial DNA among populations of *Ae. albopictus.*

Locality name	Locality ID	WJ-I	WJ-II	YC	YS	AY-I	AY-II	CC	CY	DJ	GC	GJ	GW	GY	JD	JJ	SC	SE	YJ	YE	TJ	VL
**Wonju (2017)**	WJ-I	-																				
**Wonju (2020)**	WJ-II	-0.009	-																			
**Yeoncheon**	YC	-0.051	-0.103	-																		
**Yangsan**	YS	0.043	**0.103**	0.022	-																	
**Anyang (2020)**	AY-I	0.005	0.045	0.063	0.031	-																
**Anyang (2018)**	AY-II	**0.288**	**0.375**	0.159	0.216	0.083	-															
**Chuncheon**	CC	-0.013	-0.066	0.000	0.068	0.152	**0.252**	-														
**Cheongyang**	CY	-0.266	-0.330	0.000	-0.191	-0.263	-0.160	0.000	-													
**Daejeon**	DJ	0.000	-0.054	0.000	0.085	0.186	**0.288**	0.000	0.000	-												
**Gwacheon**	GC	0.027	0.027	-0.055	0.071	-0.008	0.183	0.000	-0.313	0.020	-											
**Geoje**	GJ	**0.854**	**0.915**	**0.932**	**0.805**	**0.880**	**0.719**	**0.945**	**0.893**	**0.950**	**0.876**	-										
**Gwangju**	GW	-0.266	-0.330	0.000	-0.191	-0.263	-0.160	0.000	0.000	0.000	-0.313	**0.893**	-									
**Gyeongju**	GY	0.397	0.565	0.474	0.280	0.258	0.043	0.588	0.000	0.628	0.351	0.766	0.000	-								
**Jeung-do**	JD	0.159	**0.246**	0.167	**0.140**	0.098	0.146	**0.247**	-0.111	**0.279**	0.133	**0.826**	-0.111	0.157	-							
**Jeonju**	JJ	**0.080**	0.009	-0.108	**0.210**	0.181	**0.530**	-0.072	-0.332	-0.061	0.097	**0.954**	-0.332	0.737	**0.405**	-						
**Sokcho**	SC	-0.008	-0.028	-0.096	0.072	0.049	**0.329**	-0.055	-0.328	-0.042	0.023	**0.915**	-0.38	0.544	**0.232**	0.018	-					
**Seoul**	SE	0.024	0.117	0.189	0.023	0.014	0.019	0.300	-0.200	0.342	0.017	**0.868**	-0.200	0.152	0.077	0.311	0.121	-				
**Yeoju**	YJ	-0.140	-0.195	0.000	-0.070	-0.091	0.004	0.000	0.000	0.000	-0.167	**0.910**	0.000	0.250	0.032	-0.198	-0.191	0.000	-			
**Yeosu**	YE	-0.266	-0.330	0.000	-0.191	-0.263	-0.160	0.000	0.000	0.000	-0.313	**0.893**	0.000	0.000	-0.111	-0.332	-0.328	-0.200	0.000	-		
**JAPAN, Tokyo**	TJ	**0.633**	**0.680**	**0.662**	**0.617**	**0.649**	**0.660**	**0.677**	**0.622**	**0.684**	**0.648**	**0.847**	**0.622**	**0.498**	**0.560**	**0.733**	**0.675**	**0.649**	**0.641**	**0.622**	-	
**LAOS, Vientiane**	VL	**0.695**	**0.729**	**0.688**	**0.668**	**0.672**	**0.665**	**0.704**	**0.646**	**0.711**	**0.682**	**0.810**	**0.646**	**0.551**	**0.631**	**0.778**	**0.717**	**0.666**	**0.665**	**0.646**	**0.583**	-

Abbeviations: WJ-I= Wonju (2017), WJ-II= Wonju (2020), YC= Yeoncheon, YS= Yangsan, AY-I= Anyang (2018), AY-II= Anyang (2020), CC= Chuncheon, CY= Cheongyang, DJ= Daejeon, GC= Gwacheon, GJ= Geoje, GW= Gwangju, GY= Gyeongju, JD= Jeungdo, JJ= Jeonju, SC= Sokcho, SE= Seoul, YJ= Yeoju, YE= Yeosu, TJ= Tokyo, Japan, VL= Vientiane, Laos

Table S4 Analysis of Molecular Variance (AMOVA) by dividing Korea into A: north-south, B: east-west, C: five provincial units with concatenated sequences of mitochondrial DNA.

A: north-south

Source of variation	df	Sum of squares	Variance components	Percentage of variation (%)	F-index*
Among groups	1	1.384	-0.02047	-4.99	-0.04993
Among populations within groups	17	26.325	0.18474	45.07	**0.42923**
Within populations	129	31.689	0.24565	59.93	**0.40074**
Total	147	59.399	0.40992		

NB: North-South: North; WJ-I, WJ-II, YC, AY-I, AY-II, CC, GC, SC, SE, YJ, South; YS, CY, DJ, GJ, GW, GY, JD, JJ, YE

B: east-west

Source of variation	df	Sum of squares	Variance components	Variation (%)	F-index*
Among groups	1	1.406	-0.01871	-4.55	-0.04554

Among populations within groups	17	26.304	0.18390	44.76	**0.42812**
Within populations	129	31.689	0.24565	59.79	**0.40207**
Total	147	59.399	0.41084		

East-West: East direction, seven localities; WJ-I, WJ-II, CC, SC, GY, YS, GJ), West direction twelve localities, CY, YJ, SE, YC, JJ, JD, DJ, GC, YE, GW, AY-I, AY-II)

C: five provincial units

Source of variation	df	Sum of squares	Variance components	Variation (%)	F-index*
Among groups	4	6.977	-0.02325	-5.6	-0.05602
Among populations within groups	14	20.733	0.19262	46.41	**0.43950**
Within populations	129	31.689	0.24565	59.19	**0.40810**
Total	147	59.399	0.41502		

'df', degrees of freedom. *Significant values (P < 0.05) are with boldface after Bonferroni correction
Provincial units: Gangwon-do; WJ-I, WJ-II, CC, SC, Gyeonggi-do; AY-I, AY-II, YJ, YC, GC, SE, Gyeongsang-do; GJ, GY, YS, Jeolla-do; JD, JJ, YE, Chungcheong-do; DJ, CY

Table S5 Microsatellite genetic diversity in the 19 populations of *Ae. albopictus.*

**Locus**		**Population**																		
		TJ	AY-I	DJ	GJ	JD	CC	JJ	WJ-I	WJ-II	YC	YS	YE	YJ	SE	GC	GY	CY	SC	VL
	N	12	4	8	5	5	9	12	9	12	6	6	2	3	3	7	2	2	12	12
**Alb-tri-3**	N_a	4	2	2	-	-	-	-	-	4	-	-	3	2	-	4	3	3	4	2
	A_R	7	4	1	-	-	-	-	-	5	-	-	5	3	-	5	4	5	5	4
	H_o	0.417	0.500	0.000	-	-	-	-	-	0.583	-	-	1.000	0.333	-	0.857	1.000	1.000	0.583	0.083
	H_E	0.373	0.429	0.233	-	-	-	-	-	0.663	-	-	0.833	0.333		0.648	0.833	0.833	0.543	0.083
	F_{IS}	-0.122	-0.200	1.000	-	-	-	-	-	0.125	-	-	-0.333	0.000	-	-0.358	-0.333	-0.333	-0.077	0.000
	HWE	-	-	*	-	-	-	-	-	-	-	-	-	-	-	-	-	-	-	-
	r	1.789	1.786	1.450	1.000	1.000	1.000	1.000	1.000	2.440	1.000	1.000	3.000	1.667	1.000	2.447	3.000	3.000	2.097	1.167
**Alb-tri-45**	N_a	4	4	4	4	4	3	5	5	6	2	4	2	2	3	3	3	-	4	7
	A_R	12	8	10	7	7	4	10	4	5	3	12	2	2	11	11	6	-	6	11
	H_o	0.000	0.500	0.250	0.400	0.000	0.000	0.083	0.111	0.083	0.000	0.000	0.000	0.000	0.333	0.000	0.500	-	0.083	0.417
	H_E	0.667	0.786	0.442	0.644	0.800	0.523	0.801	0.712	0.801	0.303	0.727	0.667	0.533	0.733	0.484	0.833	-	0.591	0.823
	F_{IS}	1.000	0.400	0.451	0.407	1.000	1.000	0.900	0.852	0.900	1.000	1.000	1.000	1.000	0.600	1.000	0.500	-	0.864	0.505
	HWE	*	-	-	-	-	*	*	*	*	-	*	-	-	-	*	-	-	*	-
	r	2.435	2.857	1.949	2.462	2.929	2.073	2.938	2.664	2.982	1.576	2.697	2.000	1.933	2.600	2.010	3.000	1.000	2.235	3.079
**Alb-tri-41**	N_a	7	4	3	2	3	5	4	5	2	4	-	-	-	3	5	3	2	3	5
	A_R	22	16	14	1	15	20	16	21	2	14	-	-	-	8	19	16	15	3	18
	H_o	0.250	0.250	0.125	0.000	0.200	0.333	0.000	0.333	0.000	0.167	-	-	-	0.333	0.143	0.500	0.500	0.167	0.000
	H_E	0.812	0.750	0.492	0.533	0.378	0.621	0.652	0.765	0.507	0.742	-	-	-	0.600	0.780	0.833	0.500	0.420	0.754
	F_{IS}	0.701	0.700	0.759	1.000	0.500	0.478	1.000	0.579	1.000	0.792	-	-	-	0.500	0.829	0.500	0.000	0.614	1.000
	HWE	-	-	-	-	-	-	*	*	*	-	-	-	-	-	-	-	-	*	*
	r	3.012	2.771	1.975	1.924	1.800	2.426	2.405	2.850	1.886	2.697	1.000	1.000	1.000	2.333	2.897	3.000	2.000	1.854	2.785
**Aealbmic23**	N_a	3	2	4	-	2	2	3	5	3	2	3	2	2	2	2	-	-	-	4
	A_R	7	5	6	-	1	1	5	9	9	7	12	5	5	6	5	-	-	-	13
	H_o	0.083	0.500	0.125	-	0.000	0.000	0.083	0.222	0.250	0.333	0.833	0.500	0.333	0.000	0.143	-	-	-	0.250
	H_E	0.236	0.429	0.525	-	0.356	0.366	0.236	0.556	0.236	0.485	0.621	0.500	0.333	0.533	0.143	-	-	-	0.431
	F_{IS}	0.656	-0.200	0.774	-	1.000	1.000	0.656	0.614	-0.065	0.333	-0.389	0.000	0.000	1.000	0.000	-	-	-	0.431
	HWE	*	-	-	-	-	*	*	-	-	-	-	-	-	-	-	-	-	-	*
	r	1.478	1.786	2.147	1.000	1.667	1.673	1.478	2.250	1.478	1.857	2.232	2.000	1.667	1.933	1.286	1.000	1.000	1.000	1.914
**AealB52**	N_a	-	-	2	3	2	4	4	4	-	3	3	3	-	-	2	2	-	-	2
	A_R	-	-	10	10	10	9	9	9	-	10	9	3	-	-	1	1	-	-	1
	H_o	-	-	0.000	0.200	0.200	0.222	0.000	0.000	-	0.167	0.167	0.500	-	-	0.000	0.000	-	-	0.000
	H_E	-	-	0.233	0.600	0.200	0.595	0.435	0.732	-	0.318	0.439	0.833	-	-	0.264	0.667	-	-	0.160
	F_{IS}	-	-	1.000	0.692	0.000	0.640	1.000	1.000	-	0.500	0.643	0.500	-	-	1.000	1.000	-	-	1.000
	HWE	-	-	*	-	-	-	*	*	-	*	-	-	-	-	*	-	-	-	*
	r	1.000	1.000	1.450	2.229	1.400	2.289	1.933	2.682	1.000	1.667	1.909	3.000	1.000	1.000	1.505	2.000	1.000	1.000	1.312
**Alb-tri-20**	N_a	5	5	3	2	5	5	5	2	6	3	2	3	4	3	2	2	-	5	2
	A_R	7	8	6	6	8	9	11	8	7	4	4	6	4	4	4	3	-	6	1
	H_o	0.750	0.250	0.125	0.200	0.200	0.111	0.250	0.000	0.500	0.333	0.000	1.000	0.667	0.333	0.571	1.000	-	0.500	0.000
	H_E	0.656	0.893	0.658	0.200	0.822	0.778	0.598	0.471	0.543	0.667	0.545	0.833	0.867	0.733	0.440	0.667	-	0.819	0.464
	F_{IS}	-0.151	0.750	0.821	0.000	0.778	0.864	0.593	1.000	0.083	0.524	1.000	-0.333	0.273	0.600	-0.333	-1.000	-	0.400	1.000
	HWE	-	-	-	-	-	*	*	*	-	-	-	-	-	-	-	-	-	*	*
	r	2.427	3.357	2.387	1.400	3.062	2.865	2.360	1.833	2.204	2.404	1.939	3.000	3.200	2.600	1.789	2.000	1.000	3.027	1.822
**Alb-tri-46**	N_a	8	5	4	3	5	2	5	4	6	3	3	2	4	5	5	2	3	4	4
	A_R	14	13	6	9	7	1	13	10	10	14	10	9	11	8	8	13	8	10	4
	H_o	0.333	0.500	0.000	0.000	0.200	0.000	0.083	0.111	0.167	0.000	0.500	0.500	0.333	0.667	0.714	0.000	1.000	0.083	0.000
	H_E	0.870	0.893	0.700	0.711	0.867	0.523	0.721	0.634	0.812	0.667	0.682	0.500	0.867	0.933	0.758	0.667	0.833	0.641	0.623
	F_{IS}	0.627	0.478	1.000	1.000	0.789	1.000	0.889	0.833	0.802	1.000	0.286	0.000	0.667	0.333	0.063	1.000	-0.33	0.875	1.000

																		3		
	HWE	*	-	*	-	-	*	*	*	*	*	*	-	-	-	-	-	-	*	*
	r	3.295	3.357	2.590	2.524	3.233	1.908	2.667	2.371	3.020	2.404	2.461	2.000	3.200	3.600	2.797	2.000	3.000	2.392	2.380
**Alb-di-4**	N_a	3	2	3	3	3	3	6	4	3	3	-	-	2	3	3	3	3	5	2
	A_R	4	2	6	10	2	9	9	10	2	9	-	-	1	10	11	11	10	5	2
	H_o	0.000	0.000	0.000	0.000	0.000	0.000	0.000	0.222	0.083	0.000	-	-	0.000	0.000	0.571	1.000	0.500	0.083	0.000
	H_E	0.565	0.429	0.667	0.711	0.622	0.627	0.812	0.549	0.359	0.667	-	-	0.533	0.800	0.615	0.833	0.833	0.692	0.290
	F_{IS}	1.000	1.000	1.000	1.000	1.000	1.000	1.000	0.610	0.776	1.000	-	-	1.000	1.000	0.077	-0.333	0.500	0.884	1.000
	HWE	*	-	*	-	-	*	*	*	*	*	-	-	-	-	-	-	-	*	*
	r	2.121	1.786	2.418	2.524	2.329	2.323	3.020	2.211	1.710	2.404	1.000	1.000	1.933	2.800	2.281	3.000	3.000	2.551	1.544
**AealbD2**	N_a	2	-	4	3	2	5	6	3	6	3	5	-	3	2	4	2	3	9	7
	A_R	1	-	11	11	1	11	13	4	8	4	6	--	7	1	4	3	5	9	12
	H_o	0.000	-	0.375	0.200	0.000	0.444	0.417	0.333	0.250	0.667	0.333	-	0.000	0.000	0.143	0.500	0.500	0.500	0.500
	H_E	0.159	-	0.592	0.600	0.533	0.614	0.732	0.464	0.757	0.667	0.833	-	0.800	0.533	0.736	0.500	0.833	0.801	0.728
	F_{IS}	1.000	-	0.382	0.692	1.000	0.289	0.442	0.294	0.680	0.000	0.623	-	1.000	1.000	0.818	0.000	0.500	0.386	0.323
	HWE	*	-	-	-	-	-	-	-	-	-	-	-	-	-	-	-	-	-	-
	r	1.312	1.000	2.299	2.229	1.924	2.392	2.726	1.958	2.783	2.404	3.089	1.000	2.800	1.933	2.676	2.000	3.000	3.032	2.764
**AealbF3**	N_a	9	-	3	4	-	3	4	6	7	2	2	-	2	-	3	-	2	3	5
	A_R	14	-	3	13	-	10	5	6	10	4	1	-	1	-	2	-	2	2	8
	H_o	0.417	-	0.000	0.200	-	0.000	0.000	0.111	0.167	0.000	0.000	-	0.000	-	0.000	-	0.000	0.000	0.083
	H_E	0.616	-	0.633	0.778	-	0.392	0.623	0.817	0.761	0.303	0.545	-	0.533	-	0.703	-	0.667	0.420	0.591
	F_{IS}	0.333	-	1.000	0.765	-	1.000	1.000	0.871	0.788	1.000	1.000	-	1.000	-	1.000	-	1.000	1.000	0.864
	HWE	*	-	-	-	-	*	*	*	*	-	-	-	-	-	*	-	-	-	*
	r	2.466	1.000	2.296	2.829	1.000	1.810	2.380	3.034	2.823	1.576	1.939	1.000	1.933	1.000	2.510	1.000	2.000	1.854	2.322
**Alb-tri-18**	N_a	6	5	5	-	4	5	5	3	4	2	2	3	2	2	5	-	3	5	11
	A_R	13	15	18	-	21	28	10	20	16	16	18	14	2	6	17	-	14	13	28
	H_o	0.083	0.500	0.125	-	0.200	0.333	0.167	0.000	0.000	0.000	0.000	1.000	0.333	0.333	0.143	-	0.500	0.000	0.167
	H_E	0.750	0.786	0.775	-	0.778	0.621	0.703	0.601	0.681	0.303	0.545	0.833	0.333	0.333	0.802	-	0.833	0.826	0.920
	F_{IS}	0.893	0.400	0.848	-	0.765	0.478	0.771	1.000	1.000	1.000	1.000	-0.333	0.000	0.000	0.833	-	0.500	1.000	0.825
	HWE	*	-	*	-	-	*	*	*	*	-	-	-	-	-	-	-	-	*	*
	r	2.811	2.986	2.841	1.000	2.829	2.426	2.586	2.221	2.521	1.576	1.939	3.000	1.667	1.667	2.961	1.000	3.000	3.056	3.545
**Aealbmic21**	N_a	3	2	2	2	-	3	3	3	3	3	-	2	2	-	4	2	3	2	5
	A_R	2	2	5	4	-	3	4	4	4	6	-	2	3	-	4	3	5	2	6
	H_o	0.000	0.000	0.000	0.000	-	0.000	0.000	0.000	0.000	0.000	-	0.000	0.000	-	0.143	0.000	0.500	0.000	0.000
	H_E	0.565	0.429	0.233	0.533	-	0.523	0.652	0.627	0.420	0.667	-	0.667	0.533	-	0.780	0.667	0.833	0.464	0.783
	F_{IS}	1.000	1.000	1.000	1.000	-	1.000	1.000	1.000	1.000	1.000	-	1.000	1.000	-	0.829	1.000	0.500	1.000	1.000
	HWE	*	-	*	-	-	*	*	*	*	*	-	-	-	-	-	-	-	-	*
	r	2.121	1.786	1.450	1.924	1.000	2.073	2.356	2.323	1.854	2.404	1.000	2.000	1.933	1.000	2.840	2.000	3.000	1.822	2.876
**Alb-di-6**	N_a	7	4	4	2	3	6	5	3	6	-	2	2	3	4	3	2	2	7	6
	A_R	15	5	11	3	8	10	13	4	8	-	8	6	5	6	6	5	6	15	16
	H_o	0.417	0.001	0.125	0.000	0.400	0.000	0.083	0.000	0.167	-	0.000	0.000	0.333	0.333	0.143	0.000	0.000	0.167	0.167
	H_E	0.725	0.857	0.725	0.533	0.711	0.837	0.678	0.523	0.717	-	0.545	0.667	0.733	0.867	0.539	0.667	0.667	0.844	0.681
	F_{IS}	0.436	1.000	0.837	1.000	0.467	1.000	0.882	1.000	0.776	-	1.000	1.000	0.600	0.667	0.750	1.000	1.000	0.810	0.763
	HWE	*	-	-	-	-	*	*	*	*	-	-	-	-	-	-	-	-	*	*
	r	2.746	3.143	2.637	1.924	2.524	3.132	2.519	2.073	2.665	1.000	1.939	2.000	2.600	3.200	2.071	2.000	2.000	3.181	2.595
**Alb-tri-25**	N_a	5	2	4	3	4	6	4	8	7	-	2	2	2	2	3	2	-	7	7
	A_R	12	1	16	16	5	12	12	15	12	-	14	3	1	1	3	3	-	16	14
	H_o	0.083	0.000	0.000	0.000	0.000	0.333	0.167	0.333	0.250	-	0.167	0.000	0.000	0.000	0.000	0.000	-	0.333	0.000
	H_E	0.714	0.571	0.767	0.622	0.800	0.732	0.609	0.817	0.797	-	0.167	0.667	0.533	0.533	0.484	0.667	-	0.783	0.798
	F_{IS}	0.888	1.000	1.000	1.000	1.000	0.560	0.735	0.607	0.696	-	0.000	1.000	1.000	1.000	1.000	1.000	-	0.585	1.000
	HWE	*	-	*	-	-	-	*	*	*	-	-	-	-	-	*	-	-	*	*
	r	2.612	1.971	2.791	2.329	2.929	2.768	2.315	3.081	3.006	1.000	1.333	2.000	1.933	1.933	2.010	2.000	1.000	2.934	3.008
**Aealbmic8**	N_a	7	4	4	2	2	2	7	4	7	-	2	2	-	3	4	2	3	7	7
	A_R	13	12	6	1	4	8	16	14	9	-	6	1	-	7	7	2	6	17	14
	H_o	0.083	0.250	0.000	0.000	0.000	0.000	0.167	0.000	0.333	-	0.000	0.000	-	0.000	0.000	0.000	0.500	0.500	0.000
	H_E	0.764	0.750	0.767	0.533	0.533	0.471	0.884	0.732	0.812	-	0.485	0.667	-	0.800	0.659	0.667	0.833	0.768	0.855
	F_{IS}	0.895	0.700	1.000	1.000	1.000	1.000	0.818	1.000	0.600	-	1.000	1.000	-	1.000	1.000	1.000	0.500	0.359	1.000
	HWE	*	-	*	-	-	*	*	*	-	-	-	-	-	-	-	-	-	*	*
	r	2.890	2.771	2.791	1.924	1.924	1.833	3.343	2.682	3.022	1.000	1.857	2.000	1.000	2.800	2.501	2.000	3.000	2.866	3.214
**AealbB51**	N_a	5	-	8	5	-	8	10	9	2	5	6	2	2	2	4	4	3	6	7
	A_R	10	-	15	11	-	19	15	15	1	13	15	6	1	8	9	9	8	12	15
	H_o	0.250	-	0.625	1.000	-	0.222	0.750	1.000	0.000	0.500	0.500	0.500	0.000	0.000	0.143	1.000	1.000	0.250	0.667
	H_E	0.312	-	0.900	0.844	-	0.797	0.913	0.876	0.290	0.667	0.864	0.500	0.533	0.667	0.736	1.000	0.833	0.754	0.768
	F_{IS}	0.205	-	0.320	-0.212	-	0.733	0.185	-0.152	1.000	0.268	0.444	0.000	1.000	1.000	0.818	0.000	-0.333	0.678	0.137
	HWE	*	-	-	-	-	*	*	-	*	-	-	-	-	-	-	-	-	-	-
	r	1.667	1.000	3.435	3.133	1.000	3.036	3.507	3.326	1.544	2.566	3.253	2.000	1.933	2.000	2.676	4.000	3.000	2.782	2.866
**Total**	N_a	4.938	2.812	3.688	2.562	2.688	3.938	4.812	4.312	4.562	2.438	2.500	2.000	2.188	2.375	2.188	2.188	4.562	5.188	5.188
	A_R	10.20	7.583	9.000	7.846	7.417	10.267	10.733	10.200	7.200	8.667	9.583	5.167	3.538	6.333	7.250	6.077	7.636	8.643	10.438
	H_o	0.211	0.271	0.118	0.170	0.117	0.133	0.150	0.185	0.189	0.181	0.208	0.417	0.180	0.194	0.232	0.423	0.545	0.232	0.146
	H_E	0.549	0.500	0.584	0.490	0.463	0.564	0.628	0.617	0.572	0.403	0.438	0.510	0.467	0.504	0.598	0.594	0.531	0.585	0.609
	F_{IS}	0.650	0.630	0.810	0.743	0.828	0.789	0.784	0.731	0.700	0.685	0.664	0.487	0.733	0.759	0.631	0.522	0.385	0.663	0.769
	r	**2.261**	**2.147**	**2.307**	**2.022**	**2.034**	**2.252**	**2.471**	**2.410**	**2.309**	**1.846**	**1.912**	**2.000**	**1.962**	**2.087**	**2.329**	**2.188**	**2.188**	**2.355**	**2.450**
	Np	0.8125	0.6875	0.625	0.1875	0.25	0.625	1.0625	0.5625	0.375	0.4375	0.0625	0.125	0	0.1875	0.0625	0.0625	0	0.5625	1.25

N, Number of individuals; N_a, Number of alleles; A_R, Allelic range; H_o, observed heterozygosity; H_E, Expected heterozygosity; r, Allele richness; F_{IS}, Inbreeding Coefficient; Np, Number of private alleles HWE, Hardy–Weinberg equilibrium; *Significance of deviation from Hardy–Weinberg equilibrium at $P < 0.001$ for loci in HWE after Bonferroni correction

Abbreviations: WJ-I= Wonju (2017), WJ-II= Wonju (2020), YC= Yeoncheon, YS= Yangsan, AY-I= Anyang (2018), AY-II= Anyang (2020), CC= Chuncheon, CY= Cheongyang, DJ= Daejeon, GC= Gwacheon, GJ= Geoje, GW= Gwangju, GY= Gyeongju, JD= Jeungdo, JJ= Jeonju, SC= Sokcho, SE= Seoul, YJ= Yeoju, YE= Yeosu, TJ= Tokyo, Japan, VL= Vientiane, Laos

Table S6 Pairwise F_{ST} estimates and pairwise R_{ST} estimates for *Ae. albopictus* populations.

	TJ	AY-I	DJ	GJ	JD	CC	JJ	WJ-I	WJ-II	YC	YS	YE	YJ	SE	GC	GY	CY	SC	VL
TJ	-	**0.146**	**0.167**	**0.292**	**0.122**	**0.202**	**0.154**	**0.181**	**0.190**	**0.359**	**0.275**	**0.282**	**0.247**	**0.187**	**0.157**	**0.192**	**0.244**	**0.156**	**0.199**
AY-I	0.153	-	**0.200**	**0.360**	0.158	**0.238**	**0.179**	**0.263**	**0.265**	**0.438**	**0.355**	0.381	**0.300**	**0.207**	**0.210**	0.222	0.304	**0.187**	**0.242**
DJ	**0.539**	**0.457**	-	**0.230**	**0.201**	**0.175**	**0.175**	**0.210**	**0.211**	**0.287**	**0.258**	**0.288**	**0.267**	**0.194**	**0.210**	0.235	**0.241**	**0.222**	**0.182**
GJ	**0.678**	**0.616**	0.185	-	**0.351**	**0.278**	**0.279**	**0.260**	**0.313**	**0.421**	**0.366**	0.399	**0.395**	**0.356**	**0.287**	**0.336**	0.318	**0.299**	**0.276**
JD	0.105	0.177	**0.489**	**0.643**	-	**0.227**	**0.177**	**0.244**	**0.229**	**0.432**	**0.331**	0.366	**0.282**	0.181	**0.217**	**0.289**	0.316	**0.138**	**0.208**
CC	**0.511**	**0.501**	**0.367**	**0.480**	**0.440**	-	**0.155**	**0.208**	**0.238**	**0.306**	**0.277**	**0.318**	**0.259**	0.135	**0.205**	0.195	**0.254**	**0.222**	**0.195**
JJ	**0.248**	**0.349**	**0.442**	**0.567**	**0.183**	**0.334**	-	**0.127**	**0.229**	**0.310**	**0.258**	**0.252**	**0.213**	**0.162**	**0.149**	0.154	0.184	**0.131**	**0.149**
WJ-I	**0.332**	**0.414**	**0.406**	**0.541**	**0.274**	**0.390**	0.096	-	**0.229**	**0.351**	**0.287**	**0.252**	**0.274**	**0.224**	**0.199**	0.195	**0.236**	**0.200**	**0.204**
WJ-II	**0.142**	**0.291**	**0.619**	**0.760**	**0.145**	**0.616**	**0.344**	**0.380**	-	**0.322**	**0.284**	0.160	**0.176**	**0.174**	**0.145**	**0.205**	0.161	**0.144**	**0.212**
YC	**0.401**	**0.287**	**0.184**	**0.386**	**0.365**	**0.290**	**0.320**	**0.361**	**0.531**	-	**0.331**	**0.400**	**0.313**	**0.356**	**0.286**	**0.388**	**0.384**	**0.322**	**0.313**
YS	**0.435**	**0.302**	0.172	**0.254**	**0.329**	**0.372**	**0.376**	**0.393**	**0.512**	0.077	-	**0.282**	**0.312**	**0.303**	**0.235**	**0.357**	**0.309**	**0.265**	**0.268**
YE	**0.329**	0.125	0.200	**0.416**	0.292	**0.418**	**0.345**	0.320	**0.418**	0.180	0.089	-	0.280	0.317	**0.202**	0.286	0.302	**0.208**	**0.263**
YJ	0.198	-0.028	**0.434**	**0.607**	0.197	**0.446**	**0.288**	**0.363**	**0.349**	0.255	0.244	0.159	-	0.162	0.122	0.269	0.191	0.116	**0.238**
SE	**0.324**	**0.341**	**0.372**	**0.516**	0.259	**0.362**	**0.223**	**0.299**	**0.483**	0.202	0.111	0.336	0.334	-	**0.173**	0.206	0.238	**0.168**	**0.214**
GC	**0.278**	**0.218**	**0.429**	**0.528**	0.157	**0.379**	**0.241**	**0.331**	**0.326**	**0.274**	**0.212**	0.187	0.101	-0.040	-	0.117	0.027	**0.092**	**0.176**
GY	0.241	0.286	**0.345**	**0.513**	0.293	**0.306**	0.046	0.155	**0.480**	0.175	0.263	0.265	0.253	0.120	0.081	-	0.172	0.155	**0.210**
CY	**0.543**	0.420	**0.310**	**0.355**	0.474	**0.453**	**0.464**	0.446	**0.614**	0.309	0.100	0.137	0.440	0.256	0.200	0.376	-	0.144	**0.207**
SC	**0.220**	**0.312**	**0.585**	**0.705**	0.080	**0.521**	**0.271**	**0.349**	**0.180**	**0.480**	**0.427**	**0.390**	**0.275**	**0.259**	**0.142**	**0.372**	**0.478**	-	**0.170**
VL	**0.433**	**0.478**	**0.456**	**0.593**	**0.460**	**0.357**	**0.196**	**0.350**	**0.574**	**0.349**	**0.458**	**0.485**	**0.440**	**0.431**	**0.383**	0.139	**0.581**	**0.527**	-

Population pairwise FSTs: Distance method: Sum of squared size difference (R_{ST}) (lower diagonal), F_{ST} (upper diagonal)

Abbreviations: WJ-I= Wonju (2017), WJ-II= Wonju (2020), YC= Yeoncheon, YS= Yangsan, AY-I= Anyang (2018), AY-II= Anyang (2020), CC= Chuncheon, CY= Cheongyang, DJ= Daejeon, GC= Gwacheon, GJ= Geoje, GW= Gwangju, GY= Gyeongju, JD= Jeungdo, JJ= Jeonju, SC= Sokcho, SE= Seoul, YJ= Yeoju, YE= Yeosu, TJ= Tokyo, Japan, VL= Vientiane, Laos

Table S7 Bottleneck test for *Ae.albopictus*.

Population	M-ratio	WILCOXON TEST		
		I.A.M	T.P.M	S.M.M
TJ	**0.5367**	**0.0005**	**0.0012**	**0.0024**
AY-I	**0.4955**	**0.0007**	**0.0034**	**0.0034**
DJ	**0.4416**	0.1748	0.3203	0.5772
GJ	**0.4354**	**0.0322**	0.3750	0.6250
JD	**0.5526**	**0.0005**	**0.0010**	**0.0024**
CC	**0.4853**	**0.0137**	0.1055	0.3223
JJ	**0.4543**	**0.0061**	**0.0342**	0.1763
WJ-I	**0.4804**	**0.0161**	0.1016	0.2402
WJ-II	**0.6648**	**0.0002**	**0.0002**	**0.0002**
YC	**0.3577**	**0.0012**	**0.0012**	**0.0081**
YS	**0.3623**	**0.0031**	**0.0031**	**0.0398**
YE	**0.4847**	0.0840	0.0840	0.0840
YJ	**0.6673**	**0.0012**	**0.0012**	**0.0012**
SE	**0.4793**	**0.0068**	**0.0122**	**0.0122**
GC	**0.5414**	**0.0024**	**0.0034**	**0.0342**
GY	**0.4614**	**0.0273**	**0.0273**	**0.0273**
CY	**0.3769**	**0.0137**	**0.0137**	**0.0137**
SC	**0.6088**	**0.0024**	**0.0034**	**0.0342**
VL	**0.5569**	0.0479	0.0942	0.5879

IAM: infinite alleles model; SMM: stepwise mutation model; TPM: two phase model

Values in bold indicate a bottleneck (M-ratio ≤ 0.68; two-tailed Wilcoxon test $P < 0.05$).

Abbreviations: WJ-I= Wonju (2017), WJ-II= Wonju (2020), YC= Yeoncheon, YS= Yangsan, AY-I= Anyang (2018), AY-II= Anyang (2020), CC= Chuncheon, CY= Cheongyang, DJ= Daejeon, GC= Gwacheon, GJ= Geoje, GW= Gwangju, GY= Gyeongju, JD= Jeungdo, JJ= Jeonju, SC= Sokcho, SE= Seoul, YJ= Yeoju, YE= Yeosu, TJ= Tokyo, Japan, VL= Vientiane, Laos

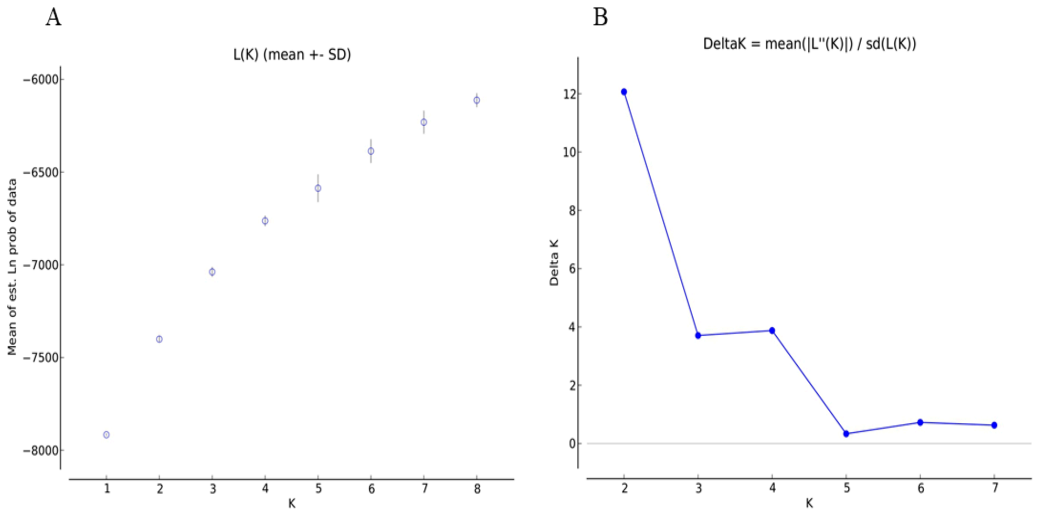

Figure S1 Estimation of the optimal number of *Aedes albopictus* populations performed using STRUCTURE software. **A** The plot of mean posterior probability (Ln $P(D)$) was based on ten repeats per cluster, K-value. **B** Optimal K-value according to maximum delta K (ΔK)

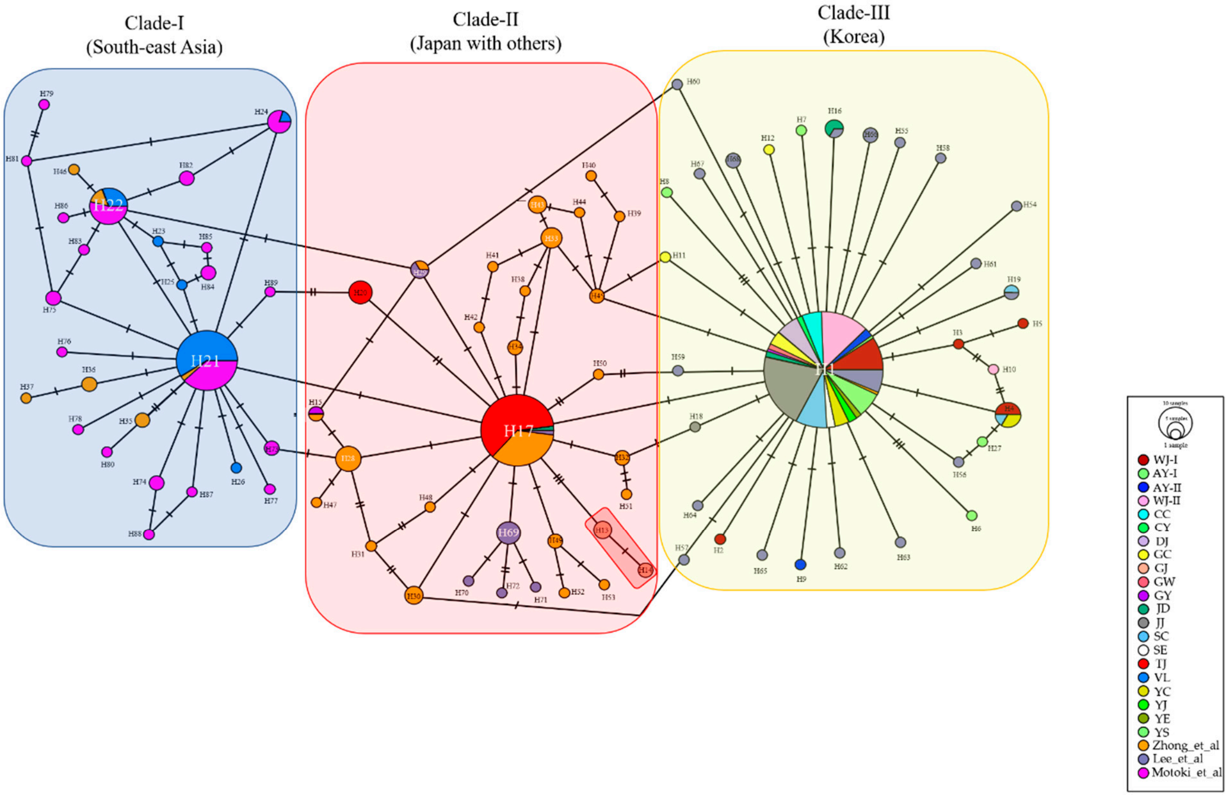

Figure S2 Minimum spanning network based on the combined NCBI-acquired *CO1* sequences and CO1 sequences from this study. It can be seen that the clades clearly distinguish between Korean and South-East Asian countries. The clade is divided into three, and some Korean populations are shared in Clade-II. The table below

shows Korean populations belonging to Clade-II in bold. (Abbreviations: WJ-I= Wonju (2017), WJ-II= Wonju (2020), YC= Yeoncheon, YS= Yangsan, AY-I= Anyang (2018), AY-II= Anyang (2020), CC= Chuncheon, CY= Cheongyang, DJ= Daejeon, GC= Gwacheon, GJ= Geoje, GW= Gwangju, GY= Gyeongju, JD= Jeungdo, JJ= Jeonju, SC= Sokcho, SE= Seoul, YJ= Yeoju, YE= Yeosu, TJ= Tokyo, Japan, VL= Vientiane, Laos).

Clades	Haplotype	No. of individuals	Haplotype distribution: Reference/region/regional code (number of populations)
Clade-I	H21	34	Laos; VL (20)<br>Zhong_et_al; Los Angeles County 2001 (1)<br>Motoki_et_al; Laos (13)
	H22	13	Laos; VL (4)<br>Zhong_et_al; Los Angeles County 2001 (2)<br>Motoki_et_al; Laos (7)
	H23	1	Laos; VL (1)
	H24	4	Laos; VL (1)<br>Motoki_et_al; Laos (4)
	H25	1	Laos; VL (1)
	H26	1	Laos; VL (1)
	H35	2	Zhong_et_al; Singapore (2)
	H36	2	Zhong_et_al; Singapore (2)
	H37	1	Zhong_et_al; Singapore (1)
	H73	2	Motoki_et_al; Laos (2)
	H74	2	Motoki_et_al; Laos (2)
	H75	2	Motoki_et_al; Laos (2)
	H76	1	Motoki_et_al; Laos (1)
	H77	1	Motoki_et_al; Laos (1)
	H78	1	Motoki_et_al; Laos (1)
	H79	1	Motoki_et_al; Laos (1)
	H80	1	Motoki_et_al; Laos (1)
	H81	1	Motoki_et_al; Laos (1)
	H82	2	Motoki_et_al; Laos (2)
	H83	1	Motoki_et_al; Laos (1)
	H84	2	Motoki_et_al; Laos (2)
	H85	1	Motoki_et_al; Laos (1)
	H86	1	Motoki_et_al; Laos (1)
	H87	1	Motoki_et_al; Laos (1)
	H88	1	Motoki_et_al; Laos (1)
	H89	1	Motoki_et_al; Laos (1)
Clade-II	H13	3	**Korea; GJ (3)**
	H14	2	**Korea; GJ (2)**
	H15	2	**Korea; GY (1)**<br>Zhong_et_al; China (1)
	H17	49	**Korea; JD (1)**<br>Japan; TJ (30)<br>Zhong_et_al; contains mainly China (1)<br>**Lee_et_al; Korea (1)**
	H20	5	Japan; TJ (5)
	H28	6	Zhong_et_al; contains mainly Los Angeles County 2001 (6)
	H29	3	Zhong_et_al; China (1)<br>**Lee_et_al; Korea (2)**
	H30	3	Zhong_et_al; China (1), Taiwan (2)
	H31	1	Zhong_et_al; China (1)
	H32	2	Zhong_et_al; China (1), Taiwan (1)
	H33	4	Zhong_et_al; China (1), Italy (2), New Jersey (1)
	H34	2	Zhong_et_al; contains mainly Singapore (2)
	H38	1	Zhong_et_al; Singapore (1)
	H39	1	Zhong_et_al; Singapore (1)
	H40	1	Zhong_et_al; Singapore (1)
	H41	1	Zhong_et_al; Italy (1)

	H42	1	Zhong_et_al; contains mainly Texas (1)
	H43	3	Zhong_et_al; Italy (1), New Jersey (2)
	H44	1	Zhong_et_al; Italy (1)
	H45	2	Zhong_et_al; Italy (1), New Jersey (1)
	H46	1	Zhong_et_al; Los Angeles County 2001 (1)
	H47	1	Zhong_et_al; Los Angeles County 2011 (1)
	H48	1	Zhong_et_al; Texas (1)
	H49	2	Zhong_et_al; Hawai'I (2)
	H50	1	Zhong_et_al; Hawai'I (1)
	H51	1	Zhong_et_al; Hawai'I (1)
	H52	1	Zhong_et_al; Hawai'I (1)
	H53	1	Zhong_et_al; Hawai'I (1)
	H69	5	**Lee_et_al; Korea (5)**
	H70	1	**Lee_et_al; Korea (1)**
	H71	1	**Lee_et_al; Korea (1)**
	H72	1	**Lee_et_al; Korea (1)**
Clade-III	H1	129	Korea;<br>WJ-I (12), WJ-II (17), AY-I (1), AY-II (3), CC (7), CY (2), DJ (8), GC (5), GW (2), GY (1), JD (2), JJ (22) SC (11), SE (3), YC (5), YJ (3), YE (2), YS (9)<br>Zhong_et_al; Japan (1)<br>Lee_et_al; Korea (8)
	H2	1	Korea; WJ-I (1)
	H3	1	Korea; WJ-I (1)
	H4	6	Korea; WJ-I (3), SC (1), YS (2)
	H5	1	Korea; WJ-I (1)
	H6	1	Korea; AY-I (1)
	H7	1	Korea; AY-I (1)
	H8	1	Korea; AY-I (1)
	H9	1	Korea; AY-II (1)
	H10	1	Korea; WJ-II (1)
	H11	1	Korea; GC (1)
	H12	1	Korea; GC (1)
	H16	3	Korea; JD (1)<br>Lee_et_al; Korea (1),
	H18	1	Korea; JJ (1)
	H19	2	Korea; SC (1)<br>Lee_et_al; Korea (1)
	H27	1	Korea; YS (1)
	H54	1	Lee_et_al; Korea (1)
	H55	1	Lee_et_al; Korea (1)
	H56	1	Lee_et_al; Korea (1)
	H57	1	Lee_et_al; Korea (1)
	H58	1	Lee_et_al; Korea (1)
	H59	1	Lee_et_al; Korea (1)
	H60	1	Lee_et_al; Korea (1)
	H61	1	Lee_et_al; Korea (1)
	H62	1	Lee_et_al; Korea (1)
	H63	1	Lee_et_al; Korea (1)
	H64	1	Lee_et_al; Korea (1)
	H65	1	Lee_et_al; Korea (1)
	H66	2	Lee_et_al; Korea (2)
	H67	1	Lee_et_al; Korea (1)
	H68	2	Lee_et_al; Korea (2)

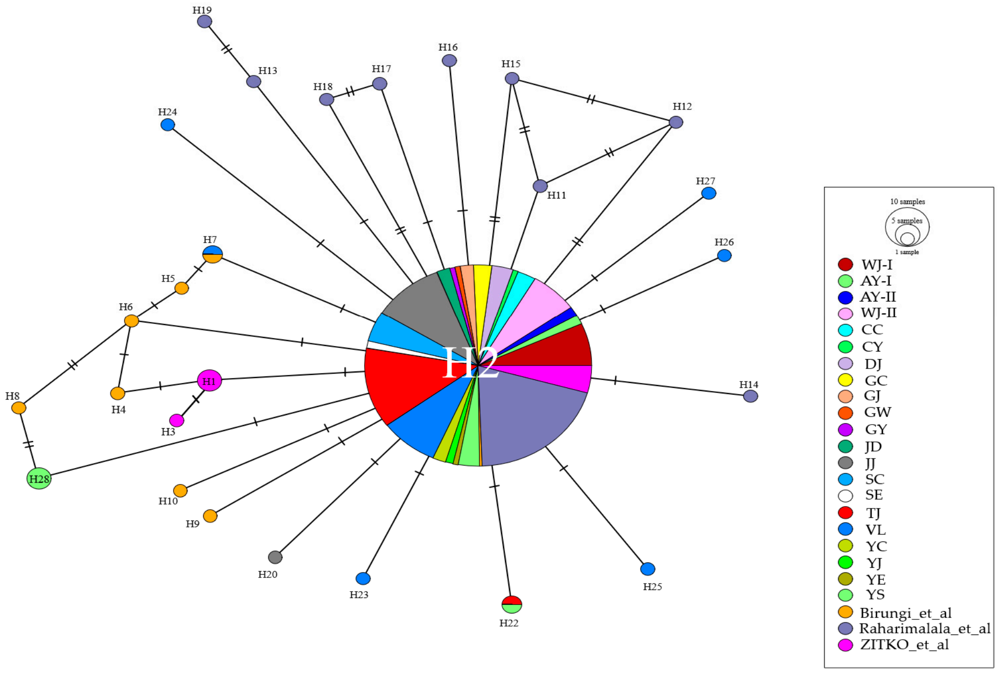

Figure S3 Minimum spanning network based on the combined NCBI-acquired *ND5* sequences and *ND5* sequences from this study. The *ND5* haplotype results shared haplotypes overall without the clear distinction from each studied country (Laos, Japan, and Korea). The table below shows the haplotype distributing to the different localities (Abbreviations: WJ-I= Wonju (2017), WJ-II= Wonju (2020), YC= Yeoncheon, YS= Yangsan, AY-I= Anyang (2018), AY-II= Anyang (2020), CC= Chuncheon, CY= Cheongyang, DJ= Daejeon, GC= Gwacheon, GJ= Geoje, GW= Gwangju, GY= Gyeongju, JD= Jeungdo, JJ= Jeonju, SC= Sokcho, SE= Seoul, YJ= Yeoju, YE= Yeosu, TJ= Tokyo, Japan, VL= Vientiane, Laos).

Haplotype	No. of individuals	Haplotype distribution: Reference/region/regional code (number of populations)
H1	3	ŽITKO_et_al; Brazil (2)<br>Birungi_et_al; Brazil (1)
H2	262	Korea;<br>WJ-I (18), WJ-II (18), AY-I (4), AY-II (4), CC (7), CY (2), DJ (8), GC (7), GJ (5), GW (2), GY (2), JD (5), JJ (27) SC (13), SE (2), YC (5), YJ (3), YE (2), YS (8)<br>ŽITKO_et_al; Viet Nam (2), USA (1), Cambodia (1), Madagascar (1), France (2), Hawai'I (1), Reunion (2)<br>Birungi_et_al; USA (1)<br>Raharimalala_et_al; Reunion (1), Madagascar (52)
H3	1	ŽITKO_et_al;
H4	1	Birungi_et_al;
H5	1	Birungi_et_al;
H6	1	Birungi_et_al;
H7	1	Birungi_et_al;
H8	1	Birungi_et_al;
H9	1	Birungi_et_al;
H10	1	Birungi_et_al;
H11	1	Raharimalala_et_al;
H12	1	Raharimalala_et_al;
H13	1	Raharimalala_et_al;
H14	1	Raharimalala_et_al;
H15	1	Raharimalala_et_al;
H16	1	Raharimalala_et_al;
H17	1	Raharimalala_et_al;
H18	1	Raharimalala_et_al;
H19	1	Raharimalala_et_al;

H20	1	Korea; JJ (1)
H21	1	Korea; SE (1)
H22	2	Korea; YS (1)<br>Japan; TJ (1)
H23	1	Laos; VL (1)
H24	1	Laos; VL (1)
H25	1	Laos; VL (1)
H26	1	Laos; VL (1)
H27	1	Laos; VL (1)
H28	1	Korea; YS (3)
